# Data sharing practices and data availability upon request differ across scientific disciplines

**DOI:** 10.1038/s41597-021-00981-0

**Published:** 2021-07-27

**Authors:** Leho Tedersoo, Rainer Küngas, Ester Oras, Kajar Köster, Helen Eenmaa, Äli Leijen, Margus Pedaste, Marju Raju, Anastasiya Astapova, Heli Lukner, Karin Kogermann, Tuul Sepp

**Affiliations:** 1 Estonian Young Academy of Sciences, Kohtu 6, 10130 Tallinn, Estonia; 2 Mycology and Microbiology Center, University of Tartu, Ravila 14a, 50411 Tartu, Estonia; 3 Institute of Chemistry, University of Tartu, Ravila 14a, 50411 Tartu, Estonia; 4 Institute of History and Archaeology, University of Tartu, Jakobi 2, 51005 Tartu, Estonia; 5 Department of Forest Sciences, University of Helsinki, PO Box 27 (Latokartanonkaari 7), Helsinki, FI-00014 Finland; 6 School of Law, University of Tartu, Näituse 20, 50409 Tartu, Estonia; 7 Institute of Education, University of Tartu, Salme 1a, 50103 Tartu, Estonia; 8 Department of Musicology, Music Pedagogy and Cultural Management, Estonian Academy of Music and Theatre, Tatari 13, 10116 Tallinn, Estonia; 9 Institute for Cultural Research and Fine Arts, University of Tartu, Ülikooli 16, 51003 Tartu, Estonia; 10 Institute of Physics, University of Tartu, W. Ostwaldi 1, 50411 Tartu, Estonia; 11 Institute of Pharmacy, University of Tartu, Nooruse 1, 50411 Tartu, Estonia; 12 Institute of Ecology and Earth Sciences, University of Tartu, Vanemuise 46, 51003 Tartu, Estonia

**Keywords:** Genetic databases, Molecular ecology

## Abstract

Data sharing is one of the cornerstones of modern science that enables large-scale analyses and reproducibility. We evaluated data availability in research articles across nine disciplines in *Nature* and *Science* magazines and recorded corresponding authors’ concerns, requests and reasons for declining data sharing. Although data sharing has improved in the last decade and particularly in recent years, data availability and willingness to share data still differ greatly among disciplines. We observed that statements of data availability upon (reasonable) request are inefficient and should not be allowed by journals. To improve data sharing at the time of manuscript acceptance, researchers should be better motivated to release their data with real benefits such as recognition, or bonus points in grant and job applications. We recommend that data management costs should be covered by funding agencies; publicly available research data ought to be included in the evaluation of applications; and surveillance of data sharing should be enforced by both academic publishers and funders. These cross-discipline survey data are available from the plutoF repository.

## Introduction

Technological advances and accumulation of case studies have led many research fields into the era of ‘big data’ - the possibility to integrate data from various sources for secondary analysis, e.g. meta-studies and meta-analyses^1,2^. Nearly half of the researchers commonly use data generated by other scientists^3^. Data sharing is a scientific norm and an important part of research ethics in all disciplines, also increasingly endorsed by publishers, funders and the scientific community^4–6^. Despite decades of argumentation^7^, much of the published data is still essentially unavailable for integration into secondary data analysis and evaluation of reproducibility, a proxy for reliability^8–10^. Furthermore, the deposited data may also be incomplete, sometimes intentionally^11–14^, e.g. in cases these exhibit mismatching sample codes or lack information about important metadata such as sex and age of studied organisms in biological and social sciences.

Although the vast majority of researchers prefer data sharing^12,15^, scientists tend to be concerned about losing their priority in future publishing and potential commercial use of their work without their consent or participation^12,16,17^. Researchers working on human subjects may be bound by legal agreements not to reveal sensitive data^16,18^. Across research fields, papers indicating available data are cited on average 25% more^19^. In research using microarrays, papers with access to raw data accumulate on average 69% more citations compared with other articles^20^. Unfortunately, higher citation rate has not motivated many researchers enough to release their data, although referees and funding agencies account for bibliometrics when evaluating researchers and their proposals^21^. Multiple case studies have revealed high variation in data availability in different journals and disciplines, ranging from 9 to 76%^8,11,13,19,22–24^. Data requests to authors are successful in 27–59% of cases, whereas the request is ignored in 14–41% cases based on previous research^10,25–28^. To promote access to data, many journals have implemented mandatory data availability statements and require data storage in supplementary materials or specific databases^29,30^. Because of poor enforcement, this has not always guaranteed access to published data because of broken links, the lack of metadata or the authors’ lack of willingness to share upon request^8,26^.

**Fig. 1:**
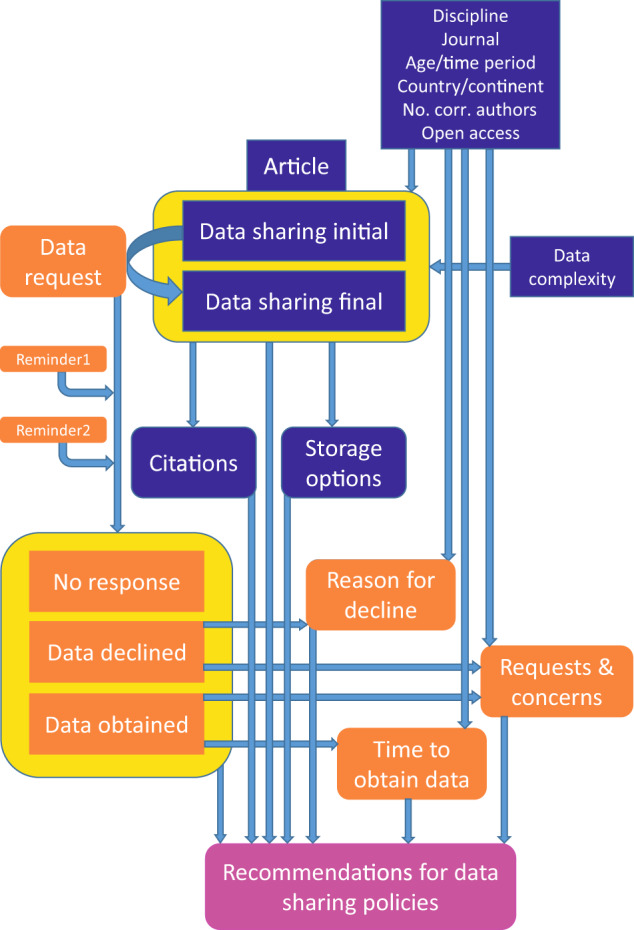
Schematic rationale of the study.

This study aims to map and evaluate cross-disciplinary differences in data sharing, authors’ concerns and reasons for denying access to data, and whether these decisions are reflected in article citations (Fig. 1). We selected the scholarly articles published in journals *Nature* and *Science* because of their multidisciplinary contents, stringent data availability policies outlined in authors’ instructions, and high-impact conclusions derived from the data of exceptional size, accuracy and/or novelty. We hypothesised that in spite of overall improvement in data sharing culture, the *actual* data availability and reasons for declining the requests to share data depend on scientific disciplines because of field-specific ‘traditions’, ‘sensitivity’ of data, or their economic potential. Our broader goal is to improve data sharing principles and policies among authors, academic publishers and research foundations.

## Results

### Initial and final data availability

**Fig. 2:**
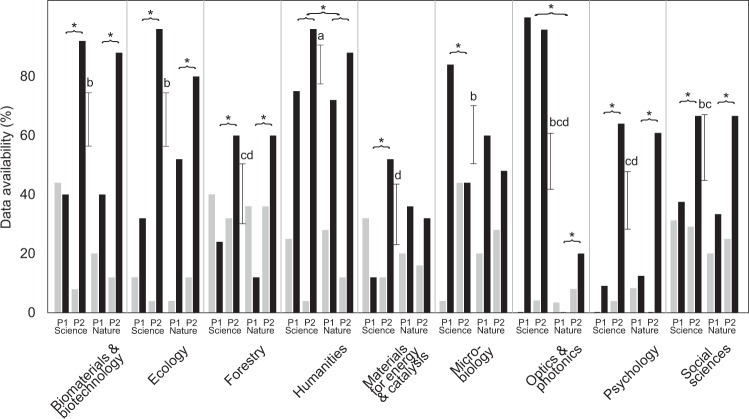
Differences in partial (grey) and full (black) data availability among disciplines depending on journal and publishing period (P1, 2000–2009; P2, 2010–2019) before contacting the authors (n = 875). Letters above bars indicate statistically significant difference groups among disciplines in full data availability compared to no data availability. Asterisks show significant differences in full data availability between journals and publishing periods.

**Fig. 3:**
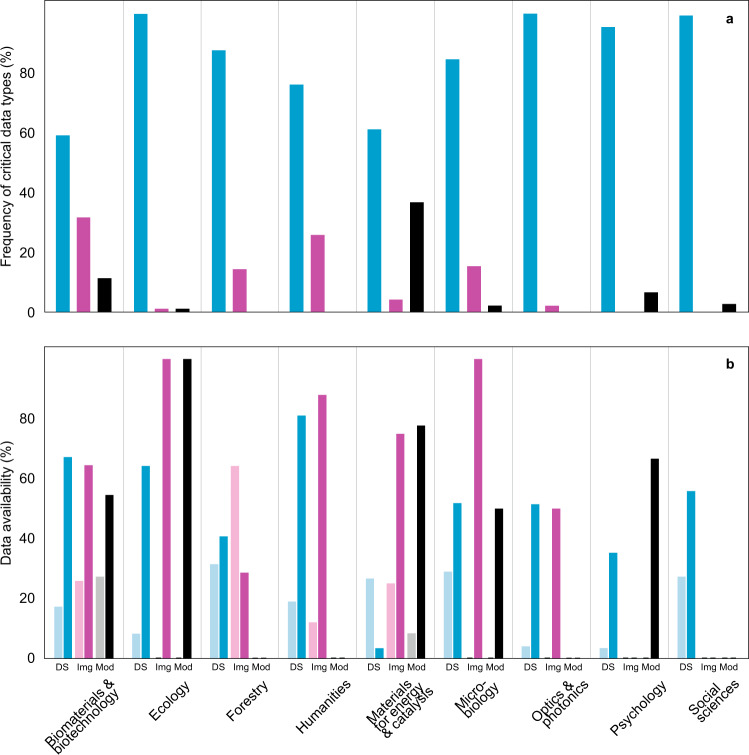
Types of critical data (n = 875). (**a**) Distribution of data types among disciplines (blue, dataset; purple, image; black, model); (**b**) Partial (light shades) and full (dark shades) data availability among disciplines depending on the type of critical data (DS, dataset; Img, image; Mod, model) before contacting the author(s).

We evaluated the availability of most critical data in 875 articles across nine scientific disciplines (Table S1) published in Nature and Science over two 10-year intervals (2000–2009 and 2010–2019) and, in case these data were not available for access, we contacted the authors. The initial (pre-contacting) full and at least partial data availability averaged at 54.2% (range across disciplines, 33.0–82.8%) and 71.8% (40.4–100.0%), respectively. Stepwise logistic regression models revealed that initial data availability differed by research field, type of data, journal and publishing period (no vs. full availability: n = 721; Somers’ D = 0.676; R^2^_model_ = 0.476; P < 0.001). According to the best model (Table S2), the data were less readily available in materials for energy and catalysis (W = 68.0; β = −1.52 ± 0.19; P < 0.001), psychology (W = 55.6; β = −1.11 ± 0.15; P < 0.001), optics and photonics (W = 18.8; β = −0.59 ± 0.14; P < 0.001) and forestry (W = 9.8; β = −0.52 ± 0.19; P = 0.002) compared with other disciplines, especially humanities (Fig. 2). Data availability was relatively lower in the period of 2000–2009 (W = 82.5; β = −0.57 ± 0.10; P < 0.001) and when the most important data were in the form of a dataset (relative to image/video and model; W = 41.5; β = −1.23 ± 0.19; P < 0.001; Fig. 3). Relatively less data were available for *Nature* (W = 32.7; β = −0.57 ± 0.19; P < 0.001), with striking several-fold differences in optics and photonics (Fig. 2).

**Fig. 4:**
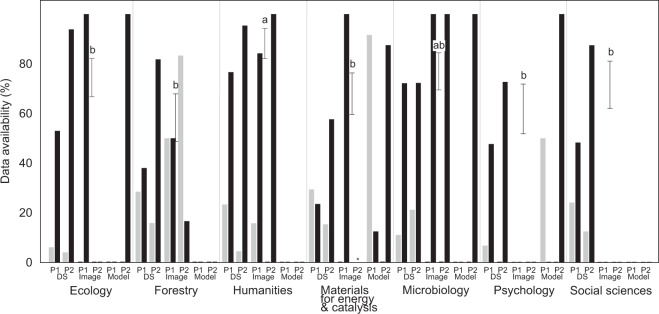
Differences in partial (grey) and full (black) data availability among disciplines after data requests (n = 672) depending on the type of critical data (DS, dataset; image; model) and publishing period (P1, 2000–2009; P2, 2010–2019). Numbers above bars indicate statistically significant difference groups among disciplines in full data availability.

**Fig. 5:**
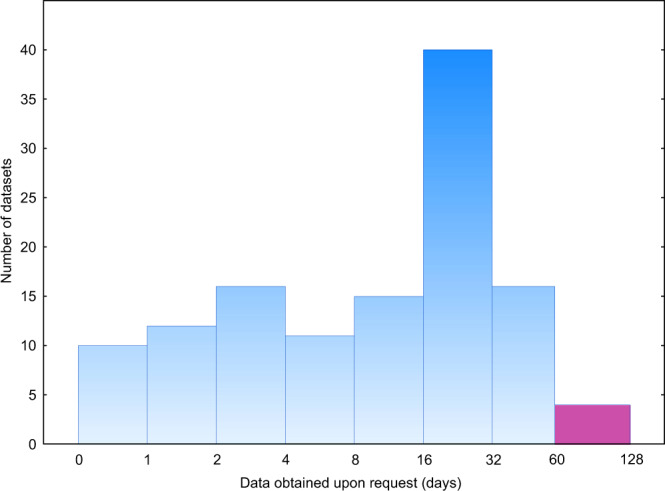
Histogram of time for receiving data from authors upon request within the 60-day reasonable time period (blue bars) and beyond (purple bar; data excluded from analyses; n = 199 requests). Note the 2-base logarithmic scale until 60 days.

Upon contacting the authors of 310 papers, the overall data availability was improved by 35.0%. Full and at least partial availability averaged 69.5% (range across disciplines, 57.0–87.9%) and 83.2% (64.9–100.0%), respectively (Fig. 4), after 60 days since contacting, a reasonable time frame^4^. The final data availability (after contacting the authors) was best predicted by scientific discipline, data type and time lapse since publishing (no vs. full availability: n = 580; D = 0.659; R^2^_model_ = 0.336; P_adj_ < 0.001; Table S2) but with no major changes in the ranking of disciplines or data types compared with the initial data availability (Fig. 4). It took a median of 15 days to receive data from the authors (Fig. 5), with a minimum time of 13 minutes. Four authors sent their data after the 60-days period since the initial request (max. 107 days). The rate of receiving data was unrelated to any studied parameter.

### Authors’ responses to data requests

The data were obtained from the authors in 39.4% of data requests on average, with a range of 27.9–56.1% among research fields. The likelihood of receiving data, the request being declined or ignored depended mostly on the time period and field of research. According to the best model (n = 310; D = 0.300; R^2^_model_ = 0.106; P_adj_ < 0.001; Table S2), the data were obtained slightly less frequently for the earlier time period (29.4% vs. 56.0%; W = 20.4; β = 0.56 ± 0.12; P_adj_ < 0.001). Receiving data upon request tended to be lowest in the field of forestry (W = 3.6; β = −0.31 ± 0.16; P_adj_ = 0.177), especially when compared with microbiology (Fig. 2).

Declining the data request averaged 19.4% and it differed most strongly among the research fields. The best model (n = 310; D = 0.508; R^2^_model_ = 0.221; P_adj_ < 0.001) revealed that the data were not made available upon request most likely in the fields of social sciences (W = 24.3; β = −1.09 ± 0.22; P_adj_ < 0.001), psychology (W = 20.0; β = −0.73 ± 0.20; P_adj_ < 0.001) and humanities (W = 5.0; β = −0.67 ± 0.30; P_adj_ = 0.078) compared with natural sciences (Fig. 2). Furthermore, the data request was more likely to be declined when the data complexity was high (W = 9.8; β = −0.59 ± 0.19; P_adj_ = 0.005), the paper was not open access in ISI Web of Science (W = 4.0; β = −0.37 ± 0.18; P_adj_ = 0.132) and published in *Science* rather than *Nature* (W = 4.6; β = −0.35 ± 0.16 P_adj_ = 0.096), although these two latter figures are non-significant when accounting for multiple testing.

**Fig. 6:**
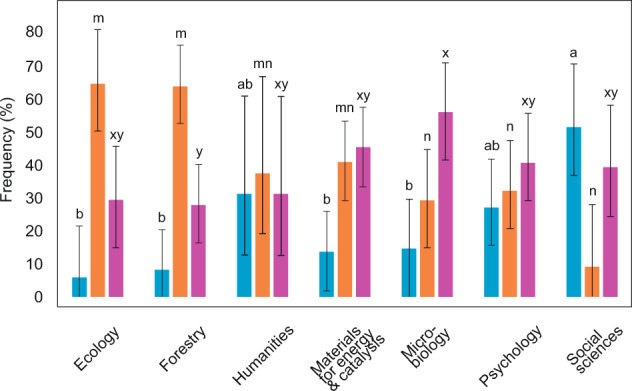
Authors’ response to data request (n = 199) depending on discipline (blue, declined; orange, ignored; purple, obtained). Bars indicate 95% CI of Sison and Glaz^51^. Letters above bars indicate statistically significant difference groups in frequency of data availability by each category based on Tukey post-hoc test and Bonferroni correction.

We received no response to 41.3% of our data requests, including two biweekly reminders. Responding to the data request differed most strongly among scientific disciplines and time periods (Fig. 6). Altogether 28.9% and 49.0% of requests were ignored by the authors of earlier (2000–2009) and later (2010 to 2019) papers, respectively. According to the best model (n = 310; D = 0.429; R^2^_model_ = 0.200; P_adj_ < 0.001; Table S2), articles from the earlier time period (W = 9.3; β = 0.41 ± 0.13; P_adj_ = 0.007) and the fields of forestry (W = 13.4; β = −0.57 ± 0.16; P_adj_ < 0.001) and ecology (W = 7.0; β = −0.53 ± 0.20; P_adj_ = 0.024) had the greatest likelihood of no response, whereas social scientists (W = 7.7; β = 0.87 ± 0.31; P_adj_ = 0.016) answered most frequently.

**Fig. 7:**
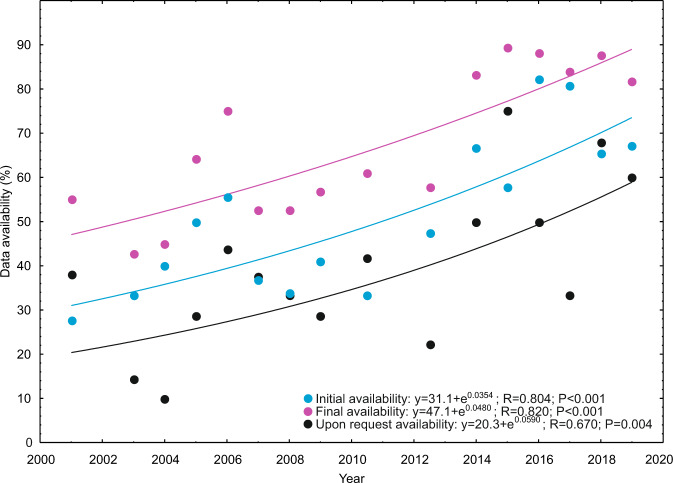
Decay in critical data availability initially (blue circles; n = 672), at the end of a 60-day contacting period (purple circles; n = 672) and upon request from the authors (black circles; n = 310).

In general, there was no residual effect of time since publication when the publication period was included in the best model. Within the 2010–2019 period, we specifically tested whether the authors publishing in 2019 and 2018 were less likely to share their data because of potential conflicting publishing interests. This hypothesis was not supported and a non-significant reverse trend was observed as the proportion of data obtained from the authors increased from 44% in 2010–2017 to 63% in 2018–2019. Accounting for time since publishing across the entire survey period, the data availability upon request decayed at a rate 5.9% year^−1^ based on an exponential model. This estimate was marginally higher than the 3.5% annual loss of publicly available data (Fig. 7). The number of articles was insufficient to test differences in data decay rates among disciplines.

### Authors’ concerns and reasons for declining data sharing

**Fig. 8:**
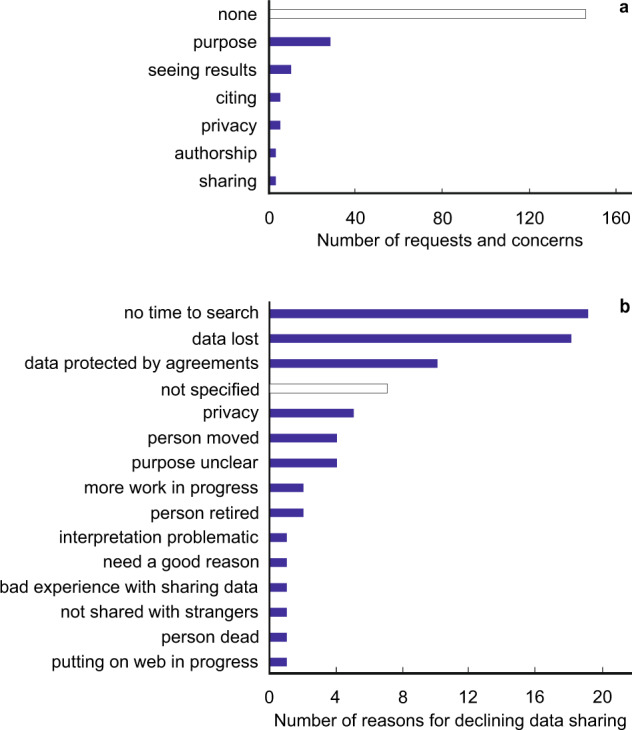
Frequency distribution of authors’ (**a**) Concerns and requests (n = 199) and (**b**) reasons for declining data sharing (n = 67). White bars indicate answers where no concerns or reasons were specified.

Upon contacting the authors, we recorded and categorised their concerns and requests related to data sharing (n = 188 authors) and their reasons for decline (n = 65). Altogether 22.9% of authors were concerned about certain aspects of our request (Fig. 8). Authors of non-open access publications (W = 4.6; β = 0.49 ± 0.23; P_adj_ = 0.064) and the field of humanities (W = 9.7; β = 1.11 ± 0.36; P_adj_ = 0.004) expressed any types of concerns or requests relatively more often (Table S2). In particular, researchers in the fields of humanities (W = 15.2; β = 1.36 ± 0.35; P_adj_ < 0.001), materials for energy and catalysis (W = 6.4; β = 0.65 ± 0.26; P_adj_ = 0.022) and ecology (W = 5.6; β = 0.81 ± 0.34; P_adj_ = 0.036) were more concerned about the study’s specific purpose than researchers on average.

Data sharing was declined by 33.0% of the 188 established contacts. When we specifically inquired about the reasons, the lack of time to search for data (29.2%), loss of data (27.7%) and privacy or legal concerns (23.1%) were most commonly indicated by the authors (Fig. 8), whereas no specific answer was provided by 10.8% of authors. According to the best binomial models (Table S2), social scientists indicated data loss more commonly than other researchers (W = 10.9; β = 1.04 ± 0.32; P_adj_ = 0.003) and psychologists pointed most commonly to legal or privacy issues (W = 4.9; β = 0.85 ± 0.38; P_adj_ = 0.078). Data decline due to legal issues became increasingly important in more recent publications (days since 01.01.2000: W = 7.2; β = 0.07 ± 0.03; P_adj_ = 0.035). The lack of time to search tended to be more common for older studies (W = 4.0; β = −0.73 ± 0.37; P_adj_ = 0.135).

### Data storage options and citations

**Fig. 9:**
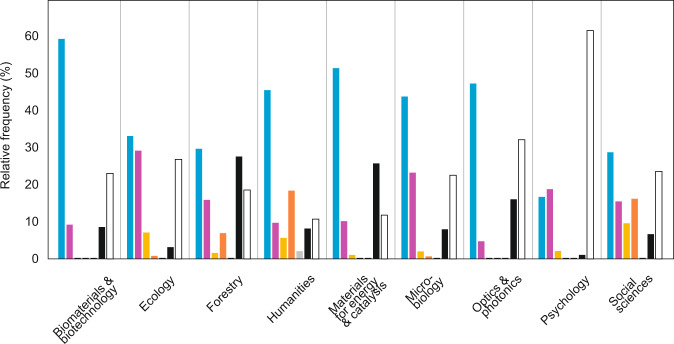
Preferred ways of data storage in articles (n = 875) representing different disciplines (blue, text and supplement; purple, data archive; yellow, authors’ homepage; vermillion, previous publications; grey, museum; black, upon (reasonable) request; white, none declared.

The ways how the data were released differed greatly among disciplines (Fig. 9), with most common storage options being the supplementary materials on the publisher’s website (62.2% of articles), various data archives (22.3%) and upon request from corresponding authors (19.7%). Although 29.8% articles declared depositing data in multiple sources, no source was indicated for 35.0% of articles. Declaring data availability upon request (n = 172) ranged from 1.0% in psychology to 52.0% in forestry, with greater frequency in earlier (days back since 31.09.2019: W = 15.0; β = 0.016 ± 0.004; P < 0.001) studies and articles by non-North American corresponding authors (by primary affiliation; W = 5.6; β = 0.23 ± 0.10; P = 0.018). With a few exceptions (three datasets only commercially available, one removed during final acceptance and one homepage corrupt), all data were successfully located for other indicated data sources, but only 42.3% of data could be obtained from the authors upon request in practice. This rate is comparable to articles with no such statement (38.3%; Chi-square test: P = 0.501).

The number of citations to articles ranged from 0.0 to 692.9 per year (median, 23.1). In contrast to the hypothesis that articles with available data accumulate more citations^20^, general linear modelling revealed no significant effect of initial or final data availability on annual citations. The model demonstrated that the average number of yearly citations was explained by research discipline (F_8,855_ = 11.2; R^2^ = 0.105; P < 0.001), data type (F_2,855_ = 7.0; R^2^ = 0.016; P < 0.001), open access status (F_1,855_ = 4.5; R^2^ = 0.005; P = 0.034) and the interaction term between open access and discipline (F_8,855_ = 2.94; R^2^ = 0.027; P = 0.003). Post-hoc tests indicated that articles with a dataset as a critical data source were cited on average 6% more than those with an image or model, and open access articles attracted 9% more citations than regular articles. Because of high variability in citation counts, it was not possible to test the interaction terms with scientific discipline in the current dataset. We speculate that the articles in *Nature* and *Science* are heavily cited on the basis of their key findings and interpretations that may mask the few extra citations raising from re-use of the data.

## Discussion

Our study uniquely points to differences among scientific disciplines in data availability as published along with the article and upon request from the authors. We demonstrate that in several disciplines such as forestry, materials for energy and catalysis and psychology, critical data are still unavailable for re-analysis or meta-analysis for more than half of the papers published in *Nature* and *Science* in the last decade. These overall figures roughly match those reported for other journals in various research fields^8,11,13,22^, but exceed the lowest reported values of around 10% available data^13,23,24^. Fortunately, data availability tends to improve, albeit slowly, in nearly all disciplines (Figs. 3, 7), which confirms recent implications from psychological and ecological journals^13,31^. Furthermore, the reverse trend we observed in microbiology corroborates the declining metagenomics sequence data availability^22^. Typically, such large DNA sequence data sets are used to publish tens of articles over many years by the teams producing these data; hence releasing both raw data and datasets may jeopardise their expectations of priority publishing. The weak discipline-specific differences among *Nature* and *Science* (Fig. 2) may be related to how certain subject editors implemented and enforced stringent data sharing policies.

After rigorous attempts to contact the authors, data availability increased by one third on average across disciplines, with full and at least partial availability reaching 70% and 83%, respectively. These figures are in the top end of studies conducted thus far^8,22^ and indicate the relatively superior overall data availability in *Science* and *Nature* compared with other journals. However, the relative rates of data retrieval upon request, decline sharing data and ignoring the requests were on par with studies covering other journals and specific research fields^10,12,25,26,28^. Across 20 years, we identified the overall loss of data at an estimated rate of 3.5% and 5.9% for initially available data and data effectively available upon request, respectively. This rate of data decay is much less than 17% year^−1^ previously reported in plant and animal sciences based on a comparable approach^24^.

While the majority of data are eventually available, it is alarming that less than a half of the data clearly stated to be available upon request could be effectively obtained from the authors. Although there may be objective reasons such as *force majeure*, these results suggest that many authors declaring data availability upon contacting may have abused the publishers’ or funders’ policy that allows statements of data availability upon request as the only means of data sharing. We find that this infringes research ethics and disables fair competition among research groups. Researchers hiding their own data may be in a power position compared with fair players in situations of big data analysis, when they can access all data (including their own), while others have more limited opportunities. Data sharing is also important for securing a possibility to re-analyse and re-interpret unexpected results^9,32^ and detect scientific misconduct^25,33^. More rigorous control of data release would prevent manuscripts with serious issues in sampling design or analytical procedures from being prepared, reviewed and eventually accepted for publication.

Our study uniquely recorded the authors’ concerns and specific requests when negotiating data sharing. Concerns and hesitations about data sharing are understandable because of potential drawbacks and misunderstandings related to data interpretation and priority of publishing^17,34^ that may outweigh the benefits of recognition and passive participation in broader meta-studies. Nearly one quarter of researchers expressed various concerns or had specific requests depending on the discipline, especially about the specific objectives of our study. Previous studies with questionnaires about hypothetical data sharing unrelated to actual data sharing reveal that financial interests, priority of additional publishing and fear of challenging the interpretations after data re-analysis constitute the authors’ major concerns^12,35,36^. Another study indicated that two thirds of researchers sharing biomedical data expected to be invited as co-authors upon use of their data^37^ although this does not fulfil the authorship criteria^6,38^. At least partly related to these issues, the reasons for declining data sharing differed among disciplines: while social scientists usually referred to the loss of data, psychologists most commonly pointed out ethical/legal issues. Recently published data were, however, more commonly declined due to ethical/legal issues, which indicates rising concerns about data protection and potential misuse. Although we offered a possibility to share anonymised data sets, such trimmed data sets were never obtained from the authors, suggesting that ethical issues were not the only reason for data decline. Because research fields strongly differed in the frequency of no response to data requests, most unanswered requests can be considered declines that avoid official replies, which may harm the authors’ reputation.

Because we did not sample randomly across journals, our interpretations are limited to the journals *Nature* and *Science*. Our study across disciplines did not account for the particular academic editor, which may have partly contributed to the differences among research fields and journals. Not all combinations of disciplines, journals and time periods received the intended 25 replicate articles because of the poor representation of certain research fields in the 2000–2009 period. This may have reduced our ability to detect statistically significant differences among the disciplines. We also obtained estimates for the final data availability for seven out of nine disciplines. Although we excluded the remaining two disciplines from comparisons of initial and final data availability, it may have slightly altered the overall estimates. The process of screening the potentially relevant articles chronologically backwards resulted in overrepresentation of more recent articles in certain relatively popular disciplines, which may have biased comparisons across disciplines. However, the paucity of residual year effect and year x discipline interaction in overall models and residual time effect in separate analyses within research fields indicate a minimal bias (Figure S1).

We recorded the concerns and requests of authors that had issues with initial data sharing. Therefore, these responses may be relatively more sceptic than the opinions of the majority of the scientific community publishing in these journals. It is likely that the authors who did not respond may have concerns and reasons for declining similar to those who refused data sharing.

Our experience shows that receiving data typically required long email exchanges with the authors, contacting other referred authors or sending a reminder. Obtaining data took on average 15 days, representing a substantial effort to both parties^39^. This could have been easily avoided by releasing data upon article acceptance. On the other hand, we received tips for analysis, caution against potential pitfalls and the authors’ informed consent upon contacting. According to our experience, more than two thirds of the authors need to be contacted for retrieving important metadata, variance estimates or specifying methods for meta-analyses^40^. Thus, contacting the authors may be commonly required to fill gaps in the data, but such extra specifications are easier to provide compared with searching and converting old datasets into a universally understandable format.

Due to various concerns and tedious data re-formatting and uploading, the authors should be better motivated for data sharing^41^. Data formatting and releasing certainly benefits from clear instructions and support from funders, institutions and publishers. In certain cases, public recognition such as badges of open data for articles following the best data sharing practices and increasing numbers of citations may promote data release by an order of magnitude^42^. Citable data papers are certainly another way forward^43,44^, because these provide access to a well-organised dataset and add to the authors’ publication record. Encouraging enlisting published data sets with download and citation metrics in grant and job applications alongside with other bibliometric indicators should promote data sharing. Relating released data in publicly available research accounts such as ORCID, ResearcherID and Google Scholar would benefit both authors, other researchers and evaluators. To account for many authors’ fear of data theft^17^ and to prioritise the publishing options of data owners, setting a reasonable embargo period for third-party publishing may be needed in specific cases such as immediate data release following data generation^45^ and dissertations.

All funders, research institutions, researchers, editors and publishers should collectively contribute to turn data sharing into a win-win situation for all parties and the scientific endeavour in general. Funding agencies may have a key role here due to the lack of conflicting interests and a possibility of exclusive allocation to depositing and publishing huge data files^46^. Funders have efficient enforcing mechanisms during reports periods, with an option to refuse extensions or approving forthcoming grant applications. We advocate that funders should include published data sets, if relevant, as an evaluation criterion besides other bibliometric information. Research institutions may follow the same principles when issuing institutional grants and employing research staff. Institutions should also insist their employees on following open data policies^45^.

Academic publishers also have a major role in shaping data sharing policies. Although deposition and maintenance of data incur extra costs to commercial publishers, they should promote data deposition in their servers or public repositories. An option is to hire specific data editors for evaluating data availability in supplementary materials or online repositories and refusing final publishing before the data are fully available in a relevant format^47^. For efficient handling, clear instructions and a machine-readable data availability statement option (with a QR code or link to the data) should be provided. In non-open access journals, the data should be accessible free of charge or at reduced price to unsubscribed users. Creating specific data journals or ‘data paper’ formats may promote publishing and sharing data that would otherwise pile up in the drawer because of disappointing results or the lack of time for preparing a regular article. The leading scientometrics platforms Clarivate Analytics, Google Scholar and Scopus should index data journals equally with regular journals to motivate researchers publishing their data. There should be a possibility of article withdrawal by the publisher, if the data availability statements are incorrect or the data have been removed post-acceptance^30^. Much of the workload should stay on the editors who are paid by the supporting association, institution or publisher in most cases. The editors should grant the referees access to these data during the reviewing process^48^, requesting them a second opinion about data availability and reasons for declining to do so. Similar stringent data sharing policies are increasingly implemented by various journals^26,30,47^.

In conclusion, data availability in top scientific journals differs strongly by discipline, but it is improving in most research fields. As our study exemplifies, the ‘data availability upon request’ model is insufficient to ensure access to datasets and other critical materials. Considering the overall data availability patterns, authors’ concerns and reasons for declining data sharing, we advocate that (a) data releasing costs ought to be covered by funders; (b) shared data and the associated bibliometric records should be included in the evaluation of job and grant applications; and (c) data sharing enforcement should be led by both funding agencies and academic publishers.

## Materials and Methods

### Data collection

To assess differences in data availability in different research disciplines, we focused our study on *Nature* and *Science*, two high-impact, general-interest journals that practise relatively stringent data availability policies^49^. Because of major changes in the public attitude and journals’ policies about data sharing, our survey was focused on two study periods, 2000–2009 and 2010–2019. We selected nine scientific disciplines as defined by the Springer Nature publishing group - biomaterials and biotechnology, ecology, forestry, humanities, materials for energy and catalysis, microbiology, optics and photonics, psychology and social sciences (see Table S1 for details) - for analysis based on their coverage in *Nature* and *Science* journals and data-driven research. These nine disciplines were selected based on the competence of our team and the objective to cover as different research fields as possible including natural sciences, social sciences and humanities. The articles were searched by discipline, keywords and/or manual browsing as follows. For *Nature*, our search was refined as https://www.nature.com/search?order=date_desc&journal=nature&article_type=research&subject=microbiology&date_range=2010-2019 (italicised parts varied). For *Science*, the corresponding search string was the following: https://search.sciencemag.org/?searchTerm=microbiology&order=newest&limit=textFields&pageSize=10&startDate=2010-01-01&endDate=2019-08-31&articleTypes=Research%20and%20reviews&source=sciencemag%7CScience. In both journals, the articles were retrieved by browsing search results chronologically backwards since September 2019 or September 2009 until reaching 25 articles matching the criteria. When the number of suitable articles was insufficient, we searched by using additional discipline-specific keywords in the title and browsed all issues manually when necessary. In some research fields, 25 articles could not be found for all journal and time period combinations and therefore, data availability was evaluated for 875 articles in total (Table S1). In each article, we identified a specific analysis or result that was critical for the main conclusion of that study based on both the authors’ emphasis and our subjective assessment. We determined whether the underlying data of these critical results - datasets, images (including videos), models (including scripts, programs and analytical procedures) or physical items - are available in the main text, supplementary materials or other indicated sources such as specific data repositories, authors’ homepages, museums, or upon request to the corresponding author (Figure S2). When available, we downloaded these data, checked for relevant metadata, identifiers and other components, and evaluated whether it is theoretically possible to repeat these specific analyses and include these materials in a field-specific metastudy. For example, in the case of a dataset, we evaluated the data table for the presence of relevant metadata and sample codes necessary to perform the analysis; for any statistical procedure, the authors must have used such a data table in their original work. We considered the data to be too raw if these either required a large amount of work (other than common data transformations) to generate the data table or model, or we had doubts whether the same data table can be reproduced with the methods described. Raw high-throughput sequencing data are typical examples of incomplete datasets, because these usually lack necessary metadata and require a thorough bioinformatics analysis, with the output depending on software and selected options. For further examples, certain optical raw images or videos make no sense without expert filtering, and computer scripts are of limited use without thorough instructions.

If these critical data were unavailable or only partly available (i.e., missing some integral metadata, instructions or explanations), we contacted the first corresponding author or a relevant author referred in relation to access to the specific item, requesting the data for a meta-study by using a pre-defined format and an institutional email address (Item S1). In the email, we carefully specified the materials required to produce a particular figure or table to avoid confusion and upsetting the authors with a messy request. We indicated that the data are intended for a metastudy in a related topic to test the authors’ willingness to share the data for actual use, not just their intention to share for no reasonable purpose. We similarly evaluated the received data for integrity and requested further information, if necessary, to meet the standards. We also recorded the responses of corresponding authors to data requests, including any specific requests or concerns and reasons for declining (Item S1).

The authors were mostly contacted early in the week and two reminders were sent ca. 14 and 28 days later if necessary (Item S1). The reminders were also addressed to other corresponding authors if relevant. If emails were returned with an error message, we contacted other corresponding authors or used an updated email address found from the internet or newer publications. We considered 60 days from sending the first email a reasonable time period for the authors to locate and send the requested data^4^.

For each article, we recorded the details of publishing (date printed, journal, discipline), corresponding authors (number, country of first affiliation, acquaintance to the contact author) and data (availability, type, ways of access)^50^. Data complexity was evaluated based on the authors’ relative amount of extra work to polish the raw data (e.g. low-complexity data include raw DNA sequence data, raw images, artefacts; high-complexity data include bioinformatics-treated molecular data sets, noise-removed images, models and scripts). As of 23.03.2020, we recorded the open access status and number of citations for each article using searches in the ISI Web of Science (https://apps.webofknowledge.com/). The citation count was expressed as citations per year, discounting the first 90 days with initially less citations.

### Data analysis

The principal aim of this study was to determine the relative importance of scientific discipline and time period on data availability and authors’ concerns in response to data sharing requests, by accounting for multiple potentially important covariates (Fig. 1). The response variables, i.e. initial and final data availability (none, partly or fully available), author’s responses (ignored, data shared or declined), concerns and reasons for decline, exhibit multinomial distribution^50^ and were hence transformed to dummy variables. Similarly, the multi-level explanatory variables (discipline, topic overlap, countries and continents of corresponding authors, data type and complexity) were transformed to dummies, whereas continuous variables (linear time, number of citations, time to obtain data, number of corresponding authors) were square root- or logarithm-transformed where appropriate. All analyses were performed in STATISTICA 12 (StatSoft Inc., Tulsa, OK, USA).

Data analysis of the dummy-transformed multinomial and binomial variables was performed using stepwise logistic regression model selection with a binomial link function using corrected Akaike information criterion (AICc) as a selection criterion, and Somers’ D statistic and model determination coefficients (R^2^) as measures of overall goodness of fit. Determination coefficients and Wald’s W statistic were used to estimate the relative importance of explanatory variables. We calculated 95% confidence intervals for multiple proportions^51^ using the R package multinomialCI (https://rdrr.io/cran/MultinomialCI/). Increasing false discovery rates related to multiple comparisons were accounted for by using Bonferroni correction of P-values (expressed as P_adj_) where appropriate.

Models with continuous response variables (proportion of available data, annual citations, time to receive data) were tested using general linear models in two steps. First, the model selection included only dummy and continuous explanatory variables. Multilevel categorical predictors corresponding to significant dummies as well as significant continuous variables were included in the final model selection as based on forward selection. To check for potential biases related to the article selection procedure in both periods, we tested the effect of discipline, period and year and all their interaction terms on initial data availability by retaining all variables in the model (Figure S1). Differences in these factor levels were tested using Tukey post-hoc tests for unequal sample size, which accounts for multiple testing issues.

## Supplementary information

Table S1

Table S2

Supplementary Information

## Data Availability

The entire dataset is available as in a spreadsheet format in plutoF data repository^50^.

## Code availability

No specific code was generated for analysis of these data.
