# Supplementary material for: Data sharing practices and data availability upon request differ across scientific disciplines
Source: Sci Data. 2021 Jul 27;8:192. doi: 10.1038/s41597-021-00981-0 (PMC8381906; doi:10.1038/s41597-021-00981-0)
Supplement: Supplementary file 3 — Supplementary Information [file 41597_2021_981_MOESM3_ESM.pdf]

Data sharing practices and data availability upon request differ across scientific disciplines

Leho Tedersoo[1,2*], Rainer Küngas[1], Ester Oras[1,3,4], Kajar Köster[1,5], Helen Eenmaa[1,6], Äli Leijen[1,7], Margus Pedaste[7], Marju Raju[1,8], Anastasiya Astapova[1,9], Heli Lukner[1,10], Karin Kogermann[1,11], Tuul Sepp[1,12]

[1]Estonian Young Academy of Sciences, Kohtu 6, 10130 Tallinn, Estonia; leho.tedersoo@ut.ee, rainerkungas@gmail.com
[2]Mycology and Microbiology Center, University of Tartu, Ravila 14a, 50411 Tartu, Estonia; leho.tedersoo@ut.ee
[3]Institute of Chemistry, University of Tartu, Ravila 14a, 50411 Tartu; ester.oras@ut.ee
[4]Institute of History and Archaeology, University of Tartu, Jakobi 2, 51005, Tartu, Estonia
[5]Department of Forest Sciences, University of Helsinki, PO Box 27 (Latokartanonkaari 7), Helsinki FI-00014, Finland; kajar.koster@helsinki.fi
[6]School of Law, University of Tartu, Näituse 20, 50409 Tartu, Estonia; helen.eenmaa@ut.ee
[7]Institute of Education, University of Tartu, Salme 1a, 50103, Tartu, Estonia; ali.leijen@ut.ee, margus.pedaste@ut.ee
[8]Department of Musicology, Music Pedagogy and Cultural Management, Estonian Academy of Music and Theatre, Tatari 13, 10116, Tallinn, Estonia; marju.raju@eamt.ee
[9]Institute for Cultural Research and Fine Arts, University of Tartu, Ülikooli 16, 51003, Tartu, Estonia; anastasiya.astapova@ut.ee
[10]Institute of Physics, University of Tartu, W. Ostwaldi 1, 50411, Tartu, Estonia; heli.valtna@ut.ee
[11]Institute of Pharmacy, University of Tartu, Nooruse 1, 50411 Tartu, Estonia; karin.kogermann@ut.ee
[12]Institute of Ecology and Earth Sciences, University of Tartu, Vanemuise 46, 51003, Tartu, Estonia; tuul.sepp@gmail.com

Supplementary material

Item S1 Page 2

Figure S1 Page 4

Figure S2 page 5

Item S1. Instructions for standardized emails when contacting the authors. XXX stands for specific information about the author, article and contacting person.

(1)

Dr. XXX,

“I am a scientist at the University of XXX working on a meta-study using multiple data sets related to your topic. To perform this study, please send me the data set that you used for running the analyses to generate Table/Figure/Model/Program script XXX, including other associated, potentially important metadata.

Please also let me know if there are any restrictions, concerns or specific requests when using these data.

Sincerely,

Dr. XXX”

(2)

IF:

1A Response and data supplied: Check data for possibility of re-analysis. See 2.

1B error message: Find a more recent email of the corresponding author (or select another corresponding author) and start over with the original data request.

1C Response but redirecting to another person: Start over with the original data request.

1D Response but worried about the lack of time to explore and analyse these data: Record this issue and reply using a standardized statement: “I only ask you to provide these data that were the basis for constructing Table/Figure/Model/Program script XXX. You must have used such a table when preparing this and it is most probably saved in the documents”. See 2.

1E Response and further questions on purpose: Record the question and clarify the purpose using a: “We will use these data in a meta-study that compiles similar data sets from multiple papers. These data will form only a small piece in a global meta-dataset and it will not be separately re-published. Unfortunately our consortium agreement asks to keep the specific topic confidential. I ensure that your data set will be appropriately cited”. See 2.

1F Response and further concerns. Record the specific concern and reply using a standardized statement: “These data will form only a small piece in a global meta-dataset and it will not be separately re-published”. Your data set will be appropriately cited”. See 2.

1G Response and request for data citation: Record the specific request and reply using a standardized statement: “Your data set will be appropriately cited”. See 2.

1H Response and request for information about the results. Record the specific request and reply using a standardized statement: “Before submission of the manuscript, I will inform you about the overall results”. See 2. [NOTE: It will be your responsibility to record the contact and provide feedback as promised. If there are multiple such requests, we can send bulk mail.]

1I Response and request for co-authorship: Record the specific request and reply using a standardized statement: “Unfortunately, we cannot grant co-authorship to all data set owners. Although we cannot accept your request, please consider sending us these data”. See 2.

1J Response and any other type of request or concern: Record the response and provide a reply so that you do not mention the intent to simply check data availability but without telling any lie. See 2.

1K Promise to send later: If no further response in TWO weeks, send a reminder. See 2.

1L No response in TWO weeks. Send a reminder. If necessary, send the second reminder in TWO weeks. See 3.

1M Declined to share: Record the response and if no reasoning is given, send a reply using a standardized question: “Can you specify the reason why you do not wish to share these data?”. See 4.

1N Points to data availability in a repository, but this turns out to be insufficient (implies a lack of willingness to help). Write a standardized statement: “I see that the very raw data are available, but its re-analysis will probably lead to different results given all analytical nuances. Therefore I ask you to kindly provide the requested elaborated dataset, which you must have used for preparation of this XXX. If this is not possible, please let me know the reason.”

1O Points that data is already in a repository in a suitable format and it was the participant’s mistake not finding this in the first place. Finish here.

1P Impolite refusal. Finish here.

1Q Does not understand what is sought. Please explain as simply as you can what exactly you want.

1R Points to the funder’s decision to keep only raw data with no possibilities of data sets. Finish here.

2. IF:

2A data are ok: Finish the topic

2B data are insufficient: ask for the missing data fields. See 3.

3. IF:

3A data are ok: Finish the topic

3B data are denied: Ask for reason if it is not specified using a standardized question: “Can you specify the reason why you do not wish to share these data?”. Record the response and finish the topic. If no response, see 3C.

3C no response: send a reminder TWO weeks after the last email. If necessary, send the second reminder in TWO weeks. See 4.

4. IF:

4A response received: document the reasons and finish the topic

4B no response after two reminders: record it and finish the topic.

(3)

When writing a reminder in 14 and 28 days later, add “REMINDER:” in front of the topic

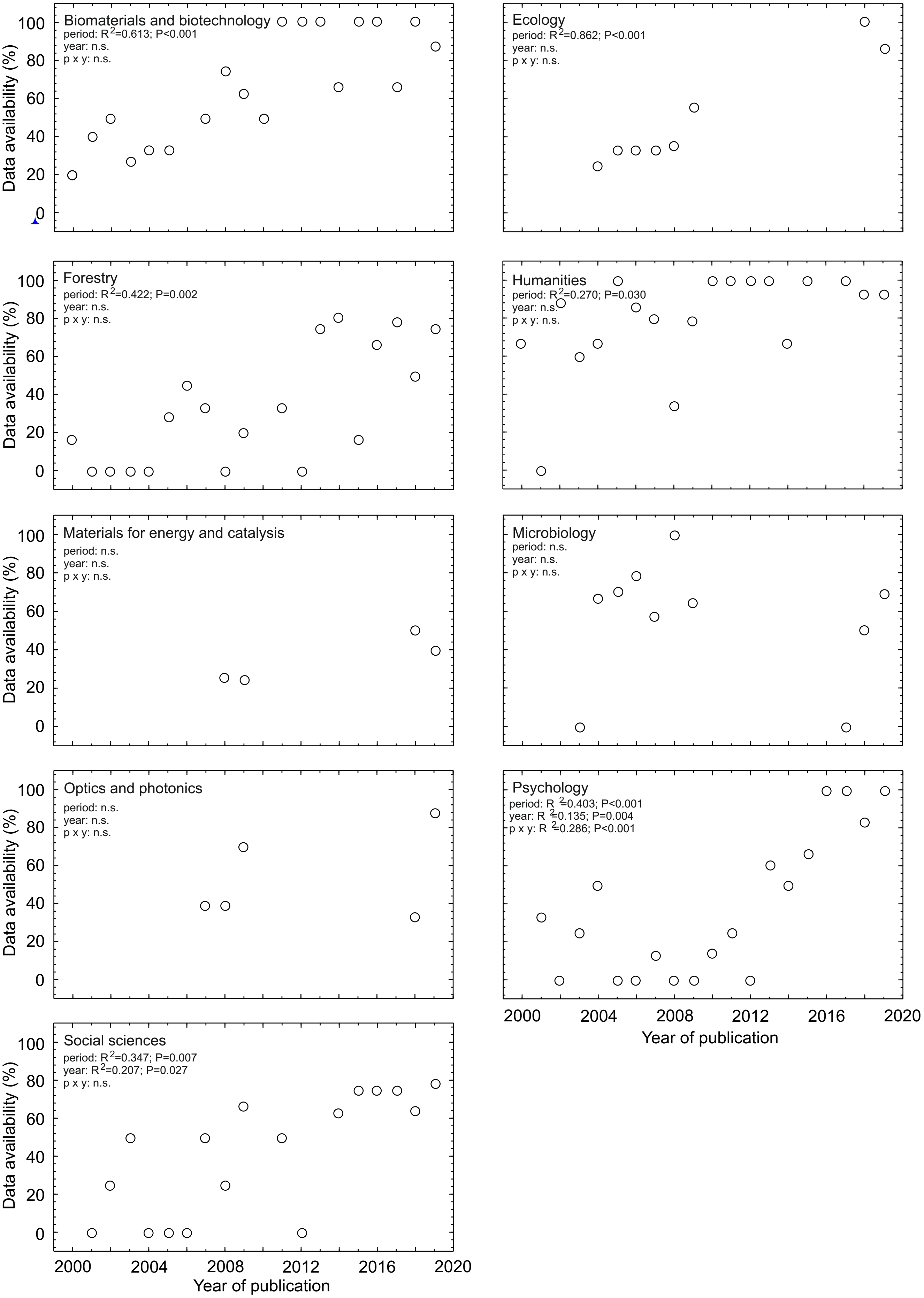

Figure S1. Temporal differences in initial data availability by discipline based on general linear models (GLMs). Relative importance of period (2000-2009 and 2010-2019), year (as a continuous variable) and their interaction (p x y) are indicated; n.s., non-significant.

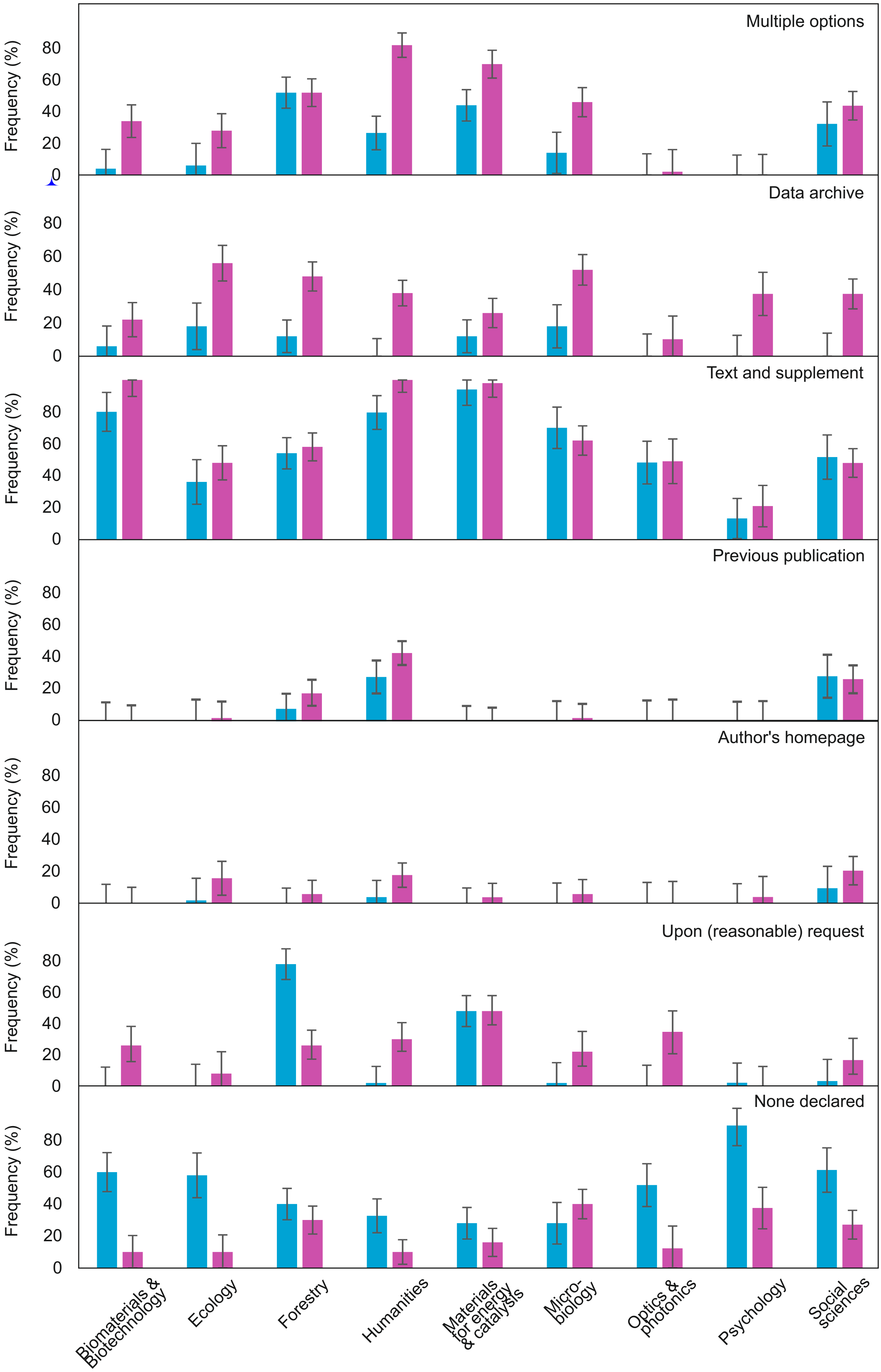

Figure S2. Frequency of declared data storage options among scientific disciplines in 2000-2009 (blue bars) and 2010-2019 (purple bars). Whiskers, 95% CI (Sison, C.P. & Glaz, J.; *J. Am. Stat. Ass.* **90**, 366-369 (1995)).
